# Morin protects the blood–brain barrier integrity against cerebral ischemia reperfusion through anti-inflammatory actions in rats

**DOI:** 10.1038/s41598-020-70214-8

**Published:** 2020-08-07

**Authors:** Satchakorn Khamchai, Wijitra Chumboatong, Janejira Hata, Chainarong Tocharus, Apichart Suksamrarn, Jiraporn Tocharus

**Affiliations:** 1 Department of Physiology, Faculty of Medicine, Chiang Mai University, Chiang Mai, 50200 Thailand; 2 Graduate School, Chiang Mai University, Chiang Mai, 50200 Thailand; 3 Center of Excellence for Innovation in Chemistry, Department of Chemistry, Faculty of Science, Ramkhamhaeng University, Bangkok, 10240 Thailand; 4 Department of Anatomy, Faculty of Medicine, Chiang Mai University, Chiang Mai, 50200 Thailand; 5 Center for Research and Development of Natural Products for Health, Chiang Mai University, Chiang Mai, 50200 Thailand

**Keywords:** Neuroscience, Physiology, Neurology

## Abstract

This study aimed to investigate the effects of morin on cerebral damage and blood–brain barrier (BBB) integrity in a middle cerebral artery occlusion (MCAO) and reperfusion model. Wistar rats were exposed to MCAO for 2 h, followed by reperfusion. Thirty mg/kg of morin was administered via intraperitoneal injection at the different time points: before ischemia, during ischemia, and at reperfusion. The rats were divided into five groups, including sham, vehicle, and three groups of morin. Twenty-four hours after reperfusion, the rats were tested for neurological deficits, and the brains were harvested to assess brain damage. In addition, brains were harvested 72 h to determine BBB disruption. We found that morin significantly reduced reactive oxygen species production and lipid peroxidation. It also decreased inflammation via reducing the expression of Toll-like receptor 4, nuclear factor kappa-beta. Morin ameliorated cerebral damage and reduced apoptosis through decreasing the cerebral infarct size, including apoptotic cell death. Moreover, morin decreased the BBB damage via reducing Evans blue extravasation, neutrophil infiltration, and increasing tight junction protein expression. Therefore, morin protected against cerebral and BBB damage by attenuating oxidative stress, inflammation, and apoptosis in MCAO and reperfusion models.

## Introduction

Stroke, the most frequent trigger of permanent disability, is the third leading cause of death in adults worldwide^1,2^. Cerebral ischemia is the most common type of stroke; it arises from a sudden interruption of the blood supply to the brain by thrombus or embolus^3,4^. Nowadays, administration of tissue-plasminogen activator (t-PA) is approved for cerebral ischemia treatment. However, to restore blood flow in the brain with t-PA would encourage the excessive production of reactive oxygen species (ROS), which would lead to cerebral ischemia/reperfusion (I/R) injury^5–7^. Moreover, ROS overexpression also triggers an inflammatory response mediated through toll-like receptor 4 (TLR4), a transmembrane protein that plays a key role in inflammation. TLR4 is expressed mainly on the membrane of neurons and glial cells to recognize the intracellular components known as damage-associated molecular patterns (DAMPs), which are released from neuronal cell damage induced by ROS^8,9^. TLR4 activation stimulates the nuclear factor kappa-beta (NF-κB) signaling pathway, which regulates the synthesis of pro-inflammatory cytokines to stimulate neuronal inflammatory such as tumor necrosis factor-α (TNF-α), interleukin-1 beta (IL-1β), inducible nitric oxide synthase (iNOS), and matrix metalloproteinase-9 (MMP-9)^10^. MMP-9 release leads to blood–brain barrier (BBB) disruption. The BBB plays a principal role in brain homeostasis and protection against various toxins. BBB disruption causes infiltration of neutrophil from blood circulation to the brain, a phenomenon that results in brain damage and leads to the high morbidity and mortality of cerebral I/R injury. Thus, ameliorating brain damage and BBB disruption following cerebral I/R would prove advantageous for the improvement of functional outcomes after stroke^11–13^.

Morin (3,5,7,2′,4′-pentahydroxyflavone) is a flavonol isolated from *Maclura cochinchinensis* (“kae lae” in Thai) that belongs to the Moraceae family. Compelling evidence has demonstrated that morin is a bioactive compound that exhibits multiple pharmacological and physiological effects, including anti-oxidant, anti-inflammatory, anti-apoptotic, and neuroprotective activity^14–18^. Moreover, morin has been reported to improve transient global cerebral ischemia model and also reduce oxidative stress, apoptosis, and inflammation in middle cerebral artery occlusion (MCAO) model^19^. However, its benefits on BBB disruption after cerebral I/R have not been reported. In the present study, we investigated the protective effects of morin during the acute phase of rats subjected to MCAO and reperfusion injury via attenuation of BBB and cerebral damage.

## Results

### Physiological parameters and regional cerebral blood flow (rCBF) monitoring during MCAO

**Figure 1:**
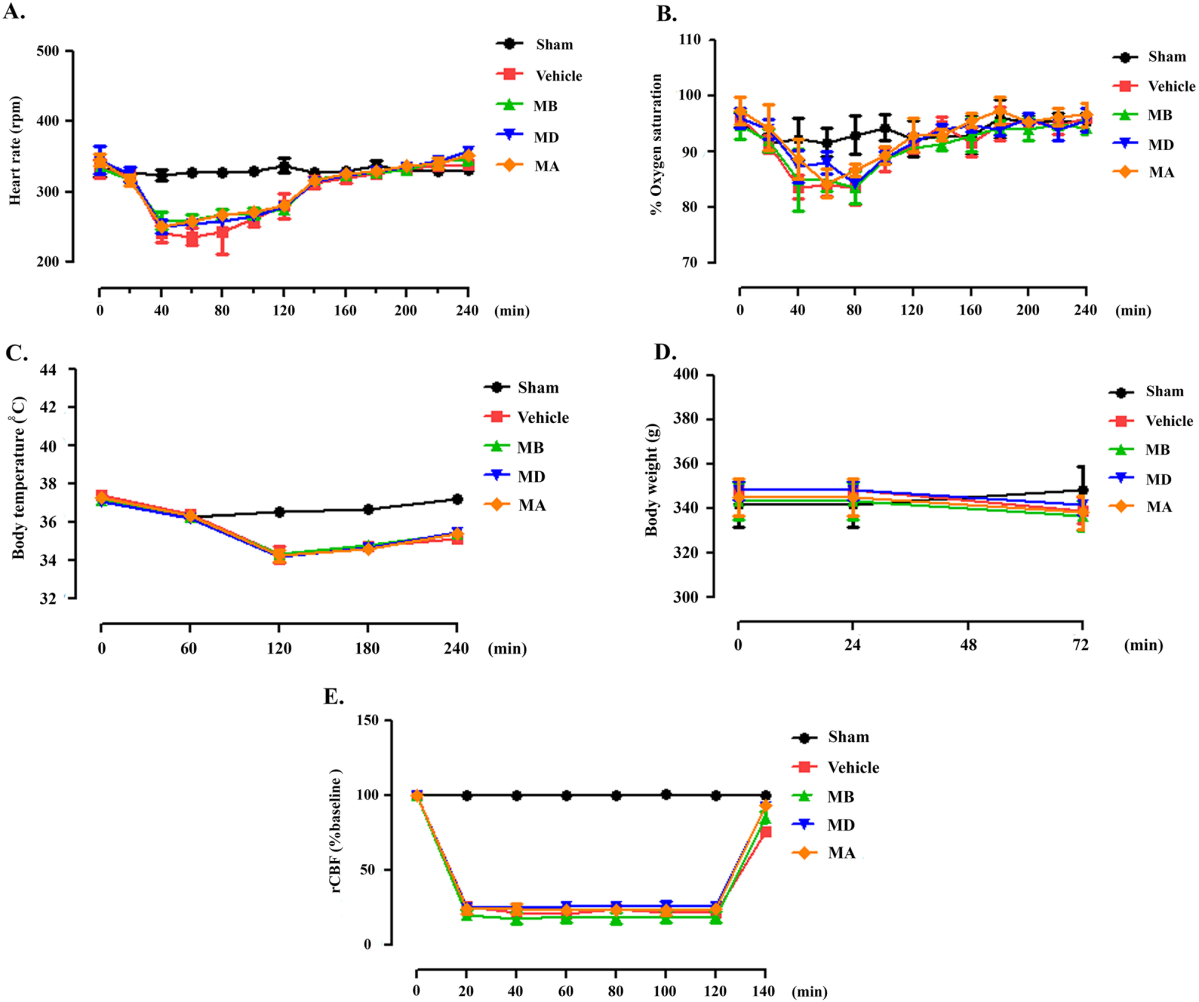
Physiological parameters during middle cerebral artery occlusion (MCAO). (**A**–**D**) Representative heart rate, oxygen saturation, body temperature, and body weight data. (**D**) Regional cerebral blood flow monitoring before ischemia, during ischemia, and after reperfusion. The data are presented as the mean ± standard error of the mean (SEM) from three independent experiments (***p < 0.001 compared with the sham group; ^###^*p* < 0.001 compared with the vehicle group; n = 6 per group).

We examined parameters during pre-ischemia, ischemia, and reperfusion. Morin treatment did not affect the rat’s body temperature, oxygen saturation, body weight, or heart rate compared with vehicle-treated rats (Fig. 1A–D). In addition, we measured rCBF (Fig. 1E). There were no changes in the sham group during the operation, but the rCBF in the MCAO rats decreased immediately to less than 25% of the baseline after occlusion (Supplementary file 1). This result showed that the MCAO model was successful.

### Morin treatment improved the neurological outcome and attenuated cerebral infarct size in rats subjected to cerebral I/R

**Figure 2:**
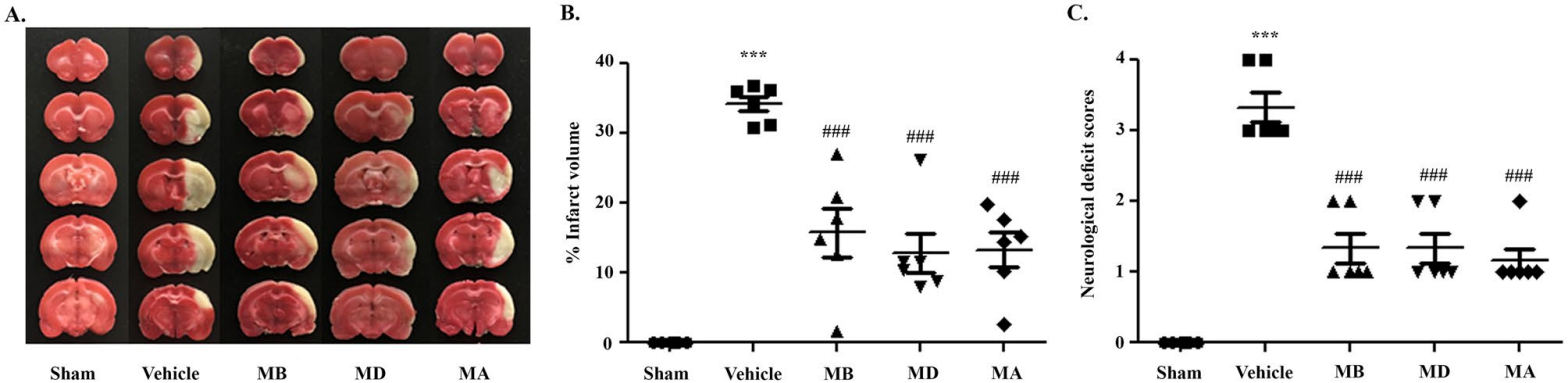
Morin treatment decreased cerebral infarction and improved neurological outcomes. (**A**) Representative images of TTC staining 24 h after reperfusion. (**B**) Percentage of infarct volume. (**C**) Neurological deficit scores 24 h after reperfusion. The data are presented as the mean ± standard error of the mean (SEM) from three independent experiments (***p < 0.001 compared with the sham group; ^###^*p* < 0.001 compared with the vehicle group; n = 6 per group).

To investigate the neuroprotective effects of morin on brain injury in MCAO rats, we first evaluated the neurological deficit scores at three different time points: before occlusion, before reperfusion, and during reperfusion. Morin-treated rats at these time points exhibited significantly reduced cerebral infarction, as indicated by decreased the infarct area and the percentage of infarct volume (Fig. 2A,B) compared with vehicle-treated rats. Moreover, morin treatment at the three different time points significantly improved neurological outcome, as indicated by increased neurological deficit scores compared with the vehicle (*p* < 0.001; Fig. 2C).

### Morin attenuated oxidative stress in cerebral I/R injury

**Figure 3:**
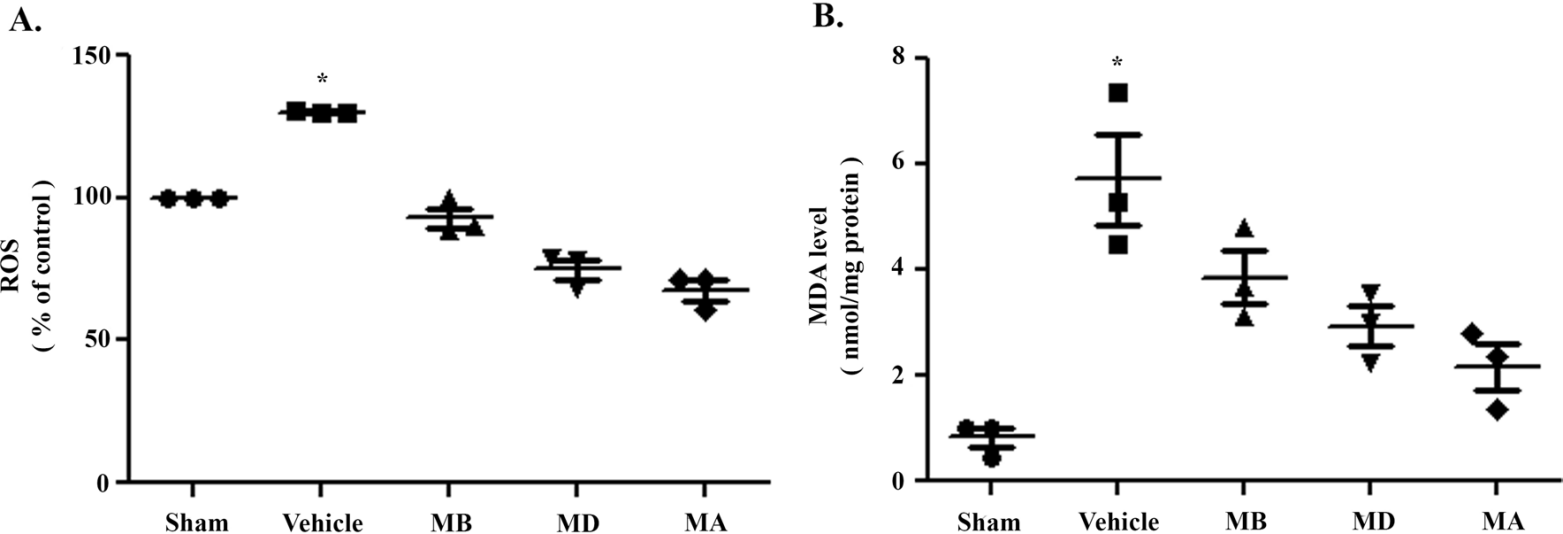
Morin treatment attenuated oxidative stress. (**A**) Percentage of reactive oxygen species (ROS) production. (**B**) Representation of malondialdehyde (MDA) level 24 h after reperfusion. The data are presented as the mean ± standard error of the mean (SEM) from three independent experiments (*p < 0.05 compared with the sham group; n = 3 each group).

To investigate whether morin reduces oxidative stress, we utilized the 2′,7′-dichlorofluorescin diacetate (DCFDA) assay and malondialdehyde (MDA) assays. Our results showed that ROS production and MDA level was significantly increased in vehicle-treated rats. However, morin administration in all groups tended to decrease, but with no significant differences when compared to I/R group (Fig. 3A,B).

### Morin decreased microglia and astrocyte expression after cerebral I/R injury

**Figure 4:**
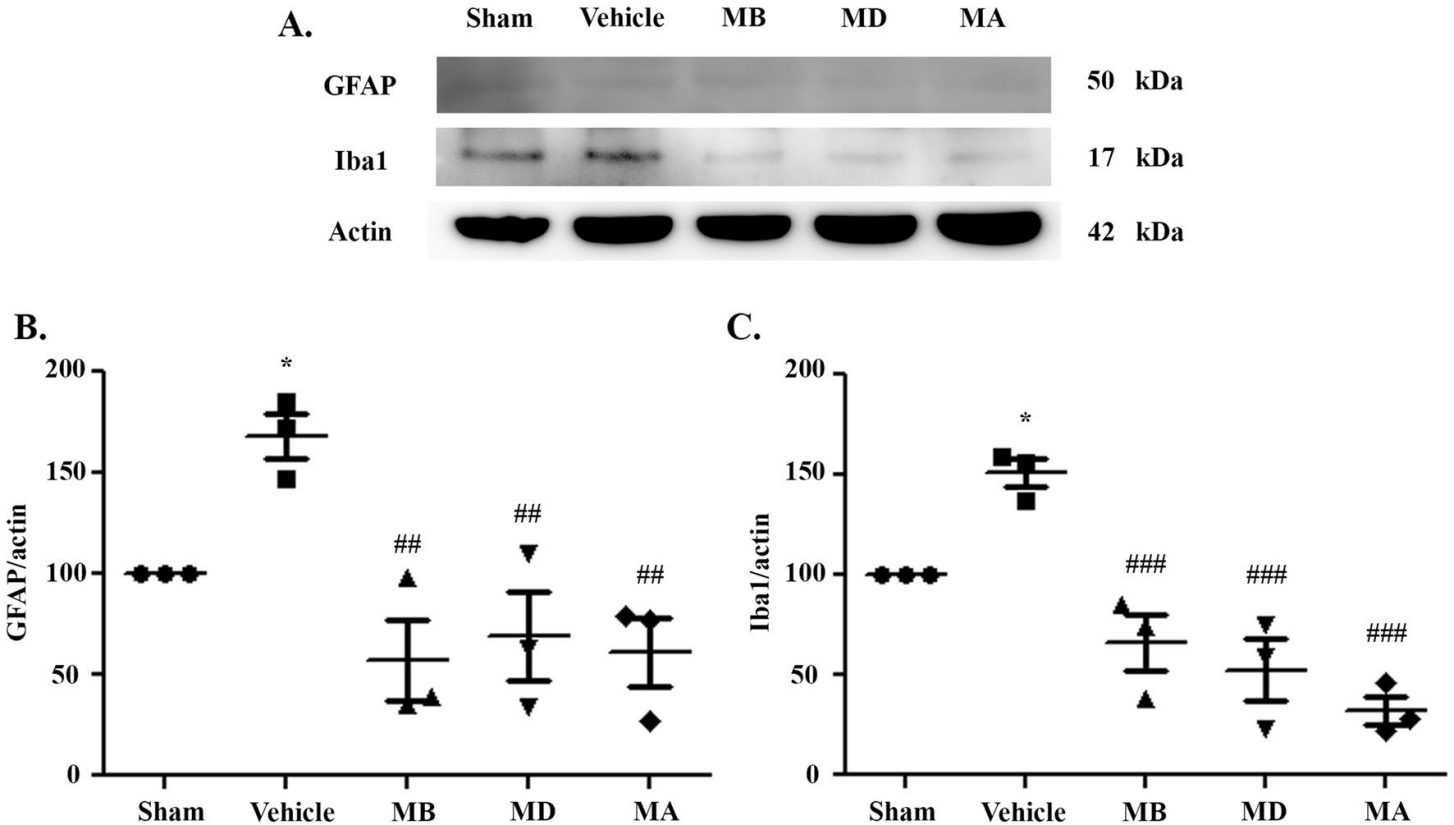
Morin decreased the expression of microglial- and astrocyte-related proteins. (**A–C**) Western blot analysis of Iba1 and GFAP in cerebral I/R models 24 h after reperfusion and quantitative analysis of the expression of the proteins normalized using β-actin. The data are presented as the mean ± standard error of the mean (SEM) from three independent experiments (*p < 0.05 compared with the sham group; ^##^*p* < 0.01 and ^###^*p* < 0.001 compared with the vehicle group; n = 3 per group).

To investigate which cells are the source of oxidative stress, we performed western blotting analysis to examine the expressions of Iba1 and glial fibrillary acidic protein (GFAP). Expression of both proteins was significantly increased in vehicle-treated rats compared to the sham group. Morin treatment significantly decreased the expression in all groups compared with vehicle group. (Fig. 4A–C). These results showed that astrocytes and microglia were the sources of ROS that led to subsequent cellular damage (e.g. lipid peroxidation).

### Morin suppressed TLR4 and inflammatory factor expression in cerebral I/R model rats

**Figure 5:**
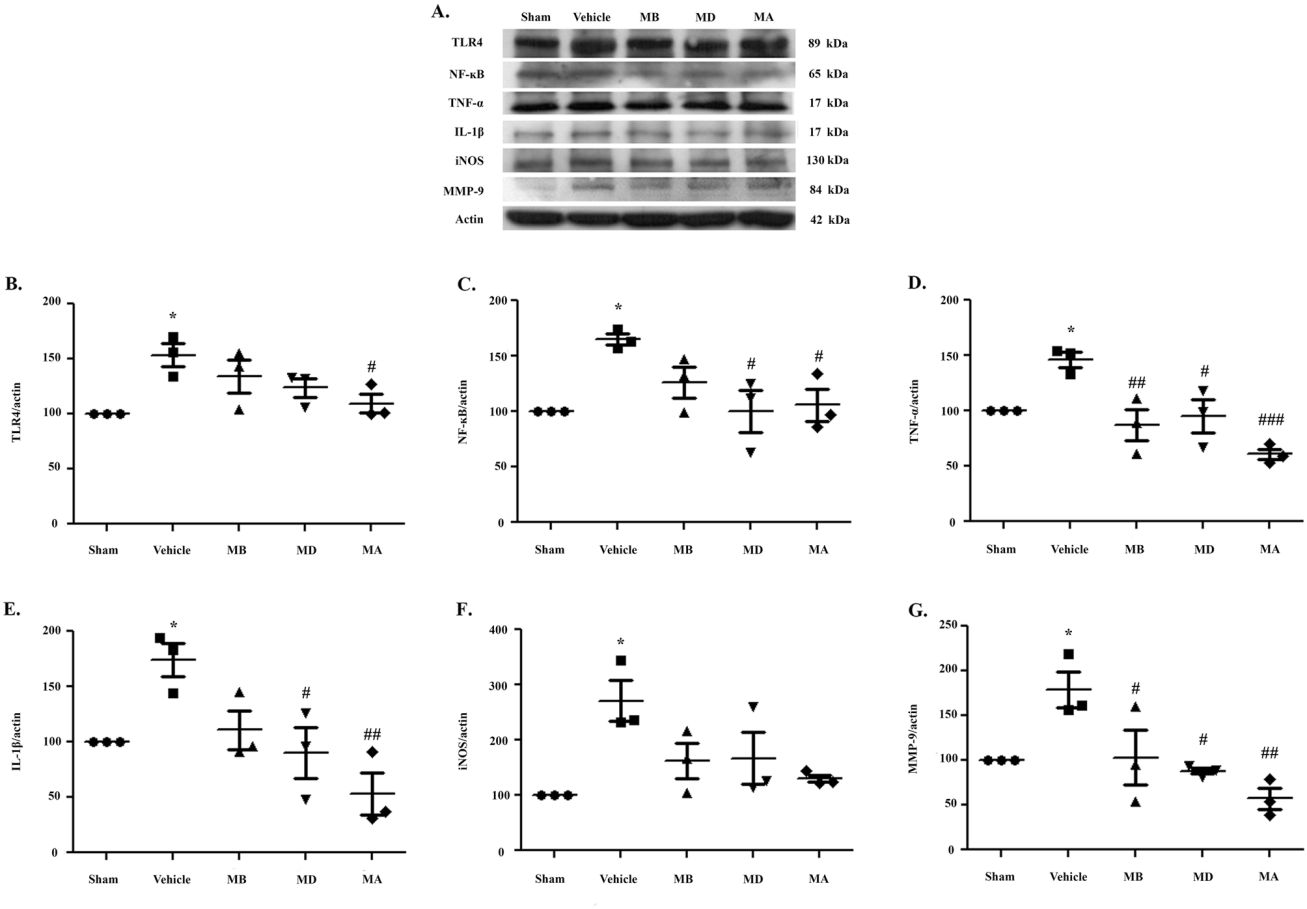
Morin suppressed TLR4 and inflammatory factor expression. (**A**) Western blot analysis of TLR4, NF-ĸB, TNF-α, IL-1β, iNOS, and MMP-9 in cerebral I/R models 24 h after reperfusion (n = 3 per group). (**B**–**G**) Quantitative analysis of protein expression normalized using β-actin. The data are presented as the mean ± standard error of the mean (SEM) from three independent experiments (*p < 0.05 and **p < 0.01 compared with the sham group; ^#^*p* < 0.05, ^##^*p* < 0.01, and ^###^*p* < 0.001 compared with the vehicle group).

To investigate the mechanism underlying the ability of morin to reduce inflammation mediated by NF-κB activation in I/R rats, we first examined the expression levels of inflammatory cytokines, including TNF-α and IL-1β, by Western blotting analysis (Fig. 5A, D, E). TNF-α and IL-1β expression in I/R rats increased compared to the control group. Treatment with morin before or during reperfusion significantly reduced the expression of both proteins compared to the I/R group treated with vehicle. The iNOS expression was significantly increased in vehicle-treated rats. However, morin administration in all groups tended to decrease, but with no significant differences when compared to I/R group (Fig. 5A,F). Next, we investigated the levels of pNF-κB by Western blotting. I/R rats exhibited significantly elevated pNF-κB expression (Fig. 5A,C). Treatment with morin before and during reperfusion significant decreased pNF-κB expression to the group treated with vehicle (Fig. 5A,C). These results indicated that morin attenuates I/R-induced NF-κB pathway activation by inhibiting proteins related to the NF-κB pathway and thereby suppressing the expressions of TNF-α and IL-1β.

We further investigated the role of morin in neuroinflammation in I/R injury to determine whether it was mediated by the TLR4 pathway. As shown in Fig. 5A,B, the I/R group presented significantly increased TLR4 expression compared to the control group. Treatment with morin during reperfusion significantly decreased TLR4 expression compared with the I/R group. However, TLR4 expression in rats treated with morin before occlusion or before reperfusion tended to decrease, but with no significant differences when compared to I/R group.

In addition, NF-ĸB activation also produces MMP-9, which is a modulator of inflammation that causes neutrophil infiltration and blood–brain barrier (BBB) breakdown^20,21^. We found that morin treatment significantly decreased MMP-9 expression compared to vehicle-treated rats (*p* < 0.05 and *p* < 0.01; Fig. 5A,G).

### Morin reduced myeloperoxidase (MPO) activity and neutrophil infiltration following cerebral I/R injury

**Figure 6:**
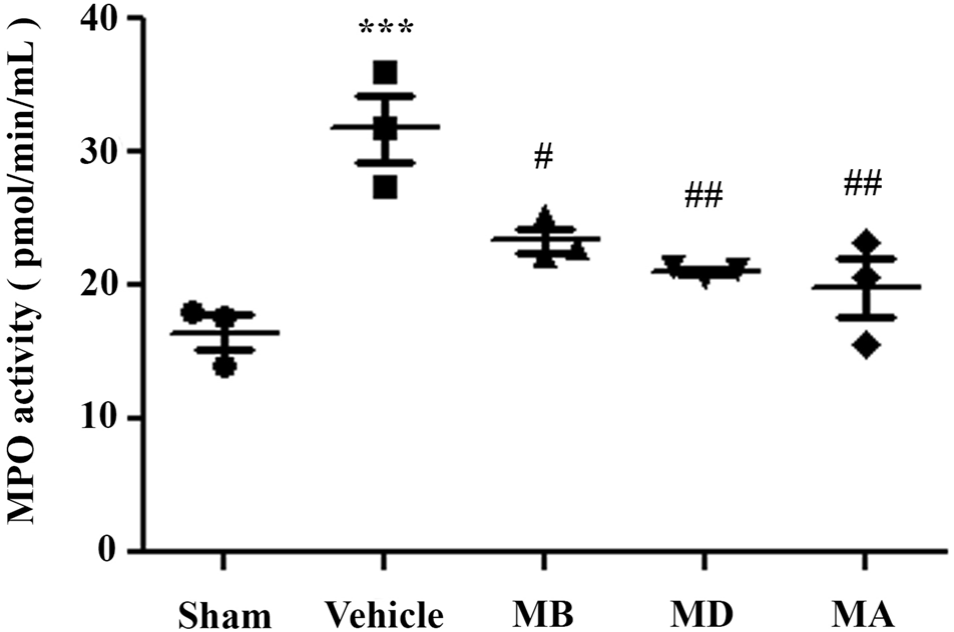
Morin reduced myeloperoxidase (MPO) activity 72 h after reperfusion (n = 3 per group). The data are presented as the mean ± standard error of the mean (SEM) from three independent experiments (***p < 0.01 compared with the sham group; ^#^*p* < 0.05 and ^##^*p* < 0.01 compared with the vehicle group).

We investigated inflammation at 72 h after reperfusion by examining neutrophil infiltration; we performed an MPO activity assay. MPO is a peroxidase enzyme that is most abundantly expressed in neutrophil granulocytes. We found that MPO activity in the vehicle group was significantly increased, but morin treatment significantly decreased this activity in all groups compared with the vehicle (*p* < 0.01 and *p* < 0.05; Fig. 6).

### Morin treatment decreased BBB leakage via improving tight junctions (TJs) of the BBB

**Figure 7:**
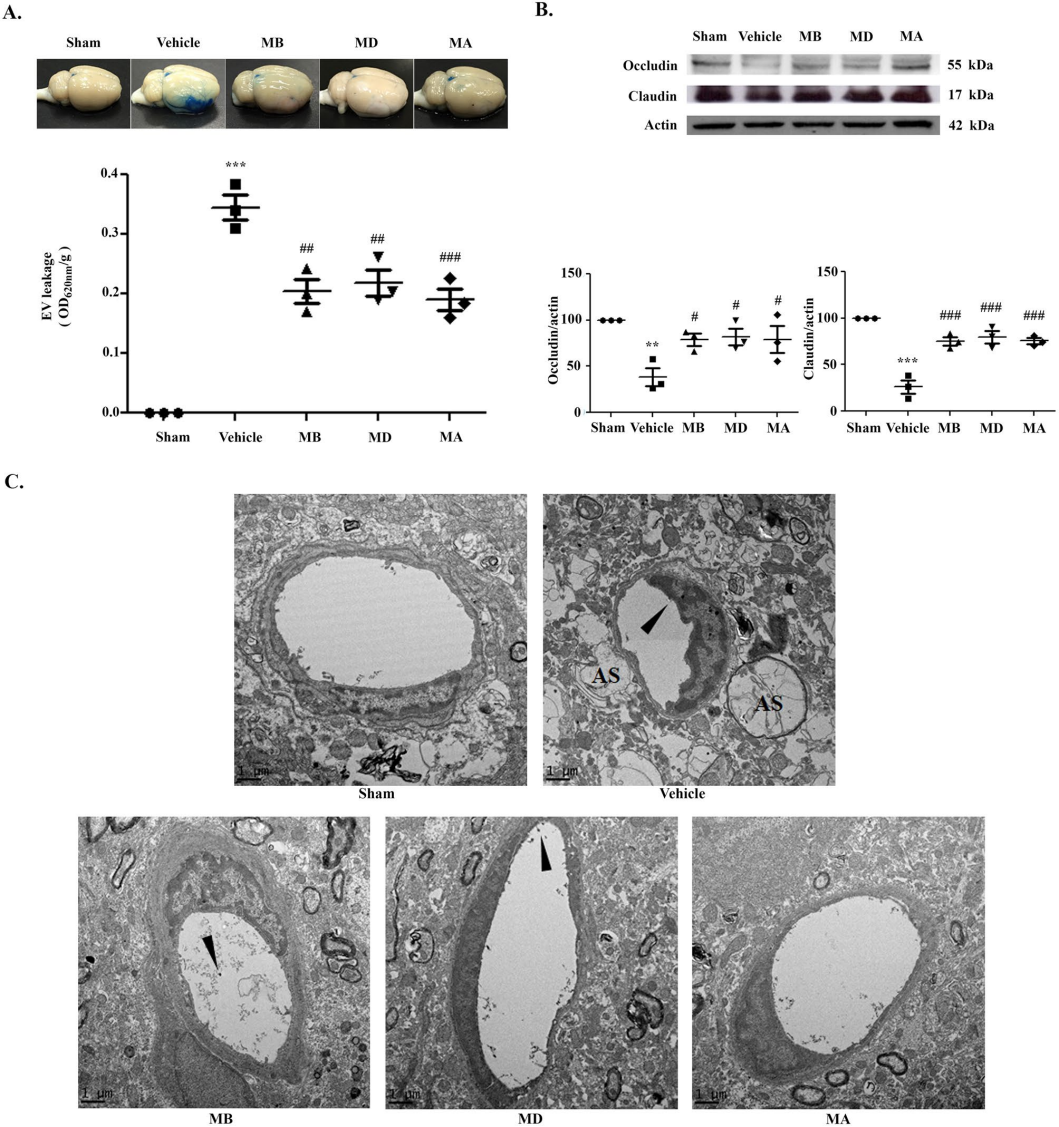
Morin improved blood–brain-barrier (BBB) disruption via increasing tight junction (TJ) proteins. (**A**) Representative images of Evans blue leakage in the brain tissues 72 h after reperfusion and representation of Evan’s blue leakage absorbance (n = 3 each group). (**B**) Western blot analysis of TJ proteins in cerebral I/R models 72 h after reperfusion and quantitative analysis of TJ protein expression normalized using β-actin (n = 3 each group). (**C**) Transmission electron micrographs (TEM) that present the ultrastructure of the BBB in cerebral I/R models 72 h after reperfusion (n = 3 per group). Arrow heads show microvillous formation. The images were visualized with TEM at a magnification of 6,000 × (scale bar = 1 µm). The data are presented as the mean ± standard error of the mean (SEM) from three independent experiments (**p < 0.01 and ***p < 0.001 compared with the sham group; ^#^*p* < 0.05, ^##^*p* < 0.01, and ^###^*p* < 0.001 compared with the vehicle group).

To investigate the effects of morin on BBB leakage in cerebral I/R models, we performed the Evans blue extravasation assay 72 h after reperfusion. The BBB leakage was represented by the seepage of the Evans blue dye as dark blue color in the brain tissue. The severity of the BBB leakage is presented as OD_620nm_/g. In the morin-treated groups, there was significantly reduced Evans blue color extravasation compared with the vehicle group (*p* < 0.001 and *p* < 0.01; Fig. 7A). As expected, the sham group had no Evans blue dye leakage.

We next detected TJ protein expression, including occluding and claudin, at 72 h after reperfusion via western blotting. We found that the vehicle group exhibited significantly reduced expression of both TJ proteins when compared to the sham group. All morin-treated groups presented significantly increased occludin and claudin expression compared with the vehicle group (*p* < 0.05 and *p* < 0.001; Fig. 7B). In addition, we used transmission electron microscopy (TEM) to examine the effects of morin on the ultrastructure of the BBB in cerebral I/R rats at 72 h after reperfusion. Our results showed that cerebral I/R caused ultrastructural changes in the BBB as well as astrocyte swelling. Moreover, there were alterations in the vascular lumen and the numbers of microvilli in the vehicle group, but these changes were attenuated in the morin-treated groups (Fig. 7C).

### Morin decreased apoptosis and reduced morphological changes

**Figure 8:**
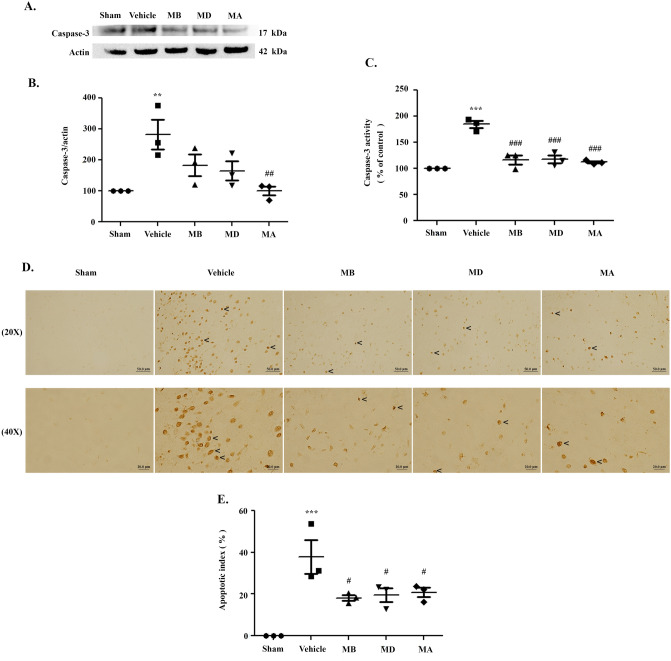

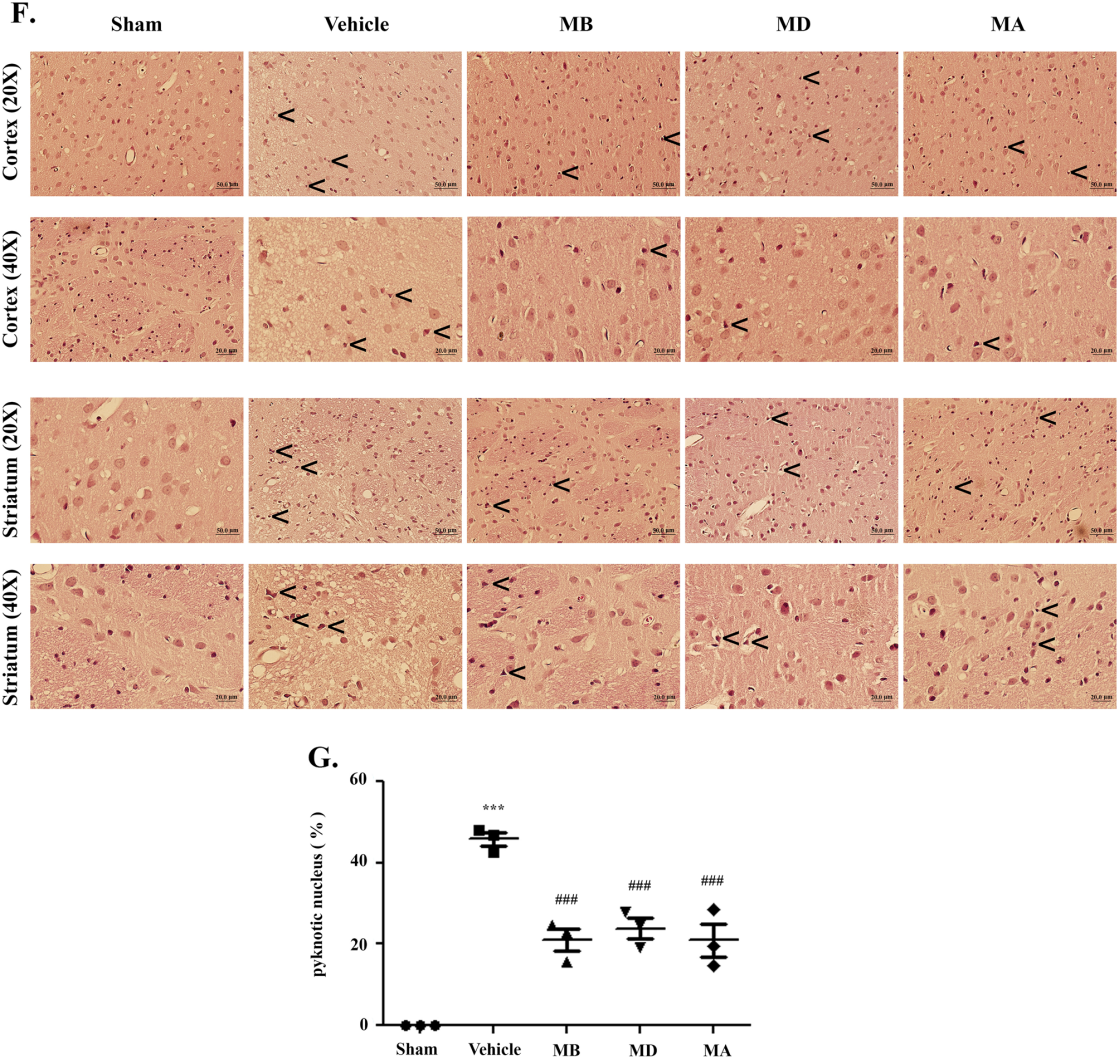
Morin decreased apoptosis and prevented morphological change. (**A**) Western blot analysis of caspase-3, an apoptotic protein, in cerebral I/R models after 24 h of reperfusion (n = 3 each group). (**B**) Quantitative analysis of caspase-3 protein expression normalized using β-actin. (**C**) Caspase-3 activity (n = 3 each group). (**D**) Representative images of TUNEL staining, shown as dark brown particles (20 × and 40 × magnification; n = 3 each group). The images were visualized with a light microscope (scale bar = 50 and 20 µm). (**E**) Representation of apoptotic index (AI). (**F**) Representative images of H&E staining of cerebral cortex and striatum (20 × and 40 × magnification; n = 3 each group). The images were visualized with a light microscope (scale bar = 50 and 20 µm). The arrow heads show the pyknotic nucleus. (**G**) Percentage of pyknotic nucleus. The data are presented as the mean ± standard error of the mean (SEM) from three independent experiments (**p < 0.01 and ***p < 0.001 compared with the sham group; ^#^*p* < 0.05, ^##^*p* < 0.01, and ^###^*p* < 0.001 compared with the vehicle group).

To investigate the effects of morin on apoptosis, we measured caspase-3 expression via western blot (Fig. 8A). Caspase-3 expression in the vehicle group was significantly increased when compared with the sham group. Morin treatment during reperfusion significantly decreased caspase-3 expression compared to the vehicle-treated rats (*p* < 0.01; Fig. 8B). We examined caspase-3 activity with a caspase-3 assay kit. This activity was significantly increased in the vehicle compared to the sham group, but morin-treated groups exhibited significantly decreased caspase-3 activity when compared with the vehicle-treated group (Fig. 8C). This result correlated with the caspase-3 expression data.

We next determined the number of apoptotic cell deaths in the penumbra area in the cerebral cortex via terminal deoxynucleotidyl transferase dUTP nick end labeling (TUNEL) staining. TUNEL-positive cells appeared as dark brown particles, which are indicative of apoptotic cells (Fig. 8D). There were no TUNEL-positive cells in the sham group. Our result showed that the apoptotic index was significantly decreased in the morin-treated compared with the vehicle group (*p* < 0.05; Fig. 8E).

After 24 h of reperfusion, we assessed morphological changes in the cerebral cortex and striatum with hematoxylin and eosin (H&E) staining. Neuronal cells with pyknotic nucleus and vacuoles around their nucleus were widely represented in the vehicle group. There were no changes in the sham group, and the morin-treated groups presented reduced changes in neuronal cells (Fig. 8F). The result showed that the percentage of pyknotic nucleus was significantly decreased in the morin-treated compared with the vehicle group (*p* < 0.001; Fig. 8G).

## Methods

### Antibodies and reagents

Anti-TLR4, anti-TNF-α, and anti-IL-1β were purchased from Abcam (Abcam, MA, USA). Anti-MMP-9 was purchased from Cell Signaling (Danvers, MA, USA). Anti-NF-κB, anti-iNOS, anti-caspase-3, anti-occludin, anti-claudin-5, anti-GFAP, anti-Iba1, and anti-actin were purchased from Millipore (Millipore, MA, USA). All other reagents were obtained from Sigma-Aldrich (St. Louis, MO).

### Extraction and isolation of morin

The dried, pulverized heartwood of *M. cochinchinensis* (5.0 kg) was extracted successively with *n*-hexane, ethyl acetate (EtOAc) and methanol (MeOH) at room temperature. The filtered solution from each extraction was evaporated to dryness under reduced pressure at 40–45 °C to give the hexane extract (6.2 g), the EtOAc extract (800.8 g), and the MeOH extract (524.3 g). To the EtOAc extract (800.5 g), MeOH–H_2_O (1:1) was added with stirring. The solid, which was separated out, was collected by filtration and recrystallized from MeOH–H_2_O (1:1) to give pure morin (5.5 g). The spectroscopic (infrared [IR], ^1^H and ^13^C nuclear magnetic resonance [NMR], 2D NMR, and mass spectra) data were in agreement with the structure of morin and were consistent with those of the reported values^22^.

### Animals and drug administration

We obtained male Wistar rats weighing 300–350 g from the National Laboratory Animal Center, Mahidol University, Salaya, Nakorn Pathom, Thailand. All animals were kept on a 12/12 h light/dark cycle with a constant ambient temperature (25 ± 1 °C). All animals were given a standard pellet rat diet and water ad libitum. All experiments in this study were approved by the Institutional Animal Care and Use Committee at the Faculty of Medicine, Chiang Mai University (Permit number: 33/2559), in compliance with National Institutes of Health (NIH) guidelines. Briefly, we randomly divided the 120 rats into five groups (n = 24) as follows: (1) Sham group; (2) MCAO group (MCAO rats treated with normal saline); (3) MB group (MCAO rats treated with morin 30 min before occlusion); (4) MD group (MCAO rats treated with morin 30 min before reperfusion); and (5) MA group (MCAO rats treated with morin at reperfusion). We chose the morin dosage, 30 mg/kg body weight (BW), based on our pilot study. We dissolved morin in dimethyl sulfoxide (DMSO) and subsequently diluted it in 2-hydroxyethyl cellulose. The vehicle group received the same volume of 2-hydroxyethyl cellulose by intraperitoneal (i.p.) injection.

### Surgical preparation of MCAO model in rats

We anesthetized rats using zoletil (30 mg/kg) and xylazine (10 mg/kg) i.p. We conducted MCAO on the rats with the use of an intraluminal monofilament technique, as previously described^23^ In brief, we identified the right common carotid artery (CCA) and external carotid artery (ECA). We introduced 4–0 filament (Doccol Corp., Sharon, MA, USA) into the internal carotid artery and advanced it until we felt a slight resistance. Importantly, we measured the sudden drop in cortical perfusion (< 25% of the baseline value) by laser Doppler flowmetry (AD instruments, Dunedin, New Zealand)^24^. After 120 min of MCAO, we carefully removed the filament to permit MCA reperfusion. After surgery, we transferred the rats to a room temperature environment (25 ± 1 °C) until sacrifice.

### Neurological assessment

Twenty-four h after reperfusion, we assessed 6 rats from each group for neurological deficits, according to a 5-point scale system, as previously described^23^. The scale is as follows: 0 = no neurological deficits; 1 = failure to extend contralateral forepaw fully; 2 = circling to the ipsilateral side when held by the tail; 3 = falling to the contralateral side; and 4 = did not walk spontaneously and has a depressed level of consciousness.

### Analysis of infarct volume

Three whole brains from each group were harvested 24 h after reperfusion, and the brain tissues were processed to six 2-mm-thick slices. We incubated the slices in 1% 2,3,5-triphenyltetrazolium chloride (TTC) at 37 °C for 20 min and then fixed them in 4% buffered formaldehyde solution overnight. We determined the infarct volume by measuring the area of the ischemic lesion in each section by using ImageJ software. The infarct volume (%) was calculated as follows: 100% × [(contralateral hemisphere volume − non-infarct ipsilateral hemisphere volume)/contralateral hemisphere volume]^25^.

### TUNEL assay

We detected apoptotic cell death by TUNEL staining on the basis of DNA fragmentation. We sacrificed rats from each group 24 h after reperfusion and fixed the brains in 4% paraformaldehyde (PFA). Next, we processed the brain tissues into 4-μm-thick slices. We deparaffinized the sections and then rehydrated them. We performed TUNEL staining according to the manufacturer’s instruction for the TUNEL assay kit (Roche Diagnostics Corp., Indianapolis, IN), with nuclei stained in brown particles. Finally, we observed the total cells and TUNEL-positive cells under a light microscope (Olympus AX70, Japan). We randomly chose five fields (× 20 and × 40 magnification) of a coronal section of the cerebral cortex at the same level and counted the number of apoptotic cells and the number of total cells of five adjacent sections^26^. The average value was used to compute the apoptotic index (Apoptosis index (AI) = number of positive cells/number of total cells).

### Investigation of BBB disruption

We investigated BBB leakage by Evans blue injection, as previously described^20^. Briefly, at 72 h after reperfusion, rats from each group were anesthetized and administered 2% Evans blue solution (4 ml/kg) via intravenous injection into the jugular vein. Thirty min after EV injection, the rats were perfused intracardially with cold phosphate-buffered saline (PBS, pH 7.4). Then, we harvested the brains, homogenized them in DMSO, and incubated the homogenates at 50 °C. We centrifuged the samples at 12,000*g* at 4 °C for 30 min and collected the supernatants. We measured the absorbance at 620 nm by using a spectrophotometer (BioTek Instruments Inc, Winooski, VT, USA).

### Histology analysis

Whole brains from each group were harvested and fixed for 48 h in 4% PFA. Next, the brain tissues were commonly embedded in paraffin, processed to 4-μm-thick slices, and stained with H&E. We observed morphological changes in the cerebral cortex and the striatum at the same level in each group were observed using a light microscope (Olympus AX70, Japan). (The percentage of pyknotic cells = (number of pyknotic nucleus/number of total cells) × 100).

### TEM

At 72 h after reperfusion, whole brains from each group were harvested and fixed with 4% PFA. Subsequently, we fixed 1 mm^3^ of the cerebral penumbra of the ischemic hemisphere with 2.5% glutaraldehyde in 0.1 phosphate buffer (pH 7.3) at 4 °C overnight. After dehydration, we saturated the samples with epoxy resin and sectioned them. We double stained the brain sections with lead citrate and uranyl acetate and then obtained images with a JEM-2200FS TEM^26^.

### Investigation of ROS production

We used oxidation-sensitive 2ʹ,7ʹ-dichlorofluorescein diacetate (DCFH-DA) dye to investigate intracellular ROS. Twenty-four h after reperfusion, we harvested the cerebral penumbra from each group and processed them to 2-mm-thick slices at the same level. We added total lysis buffer with protease inhibitor cocktail, including 10 mM HEPES (pH 7.9), 1.5 mM MgCl_2_ and 10 mM KCl. We homogenized the samples, centrifuged them at 12,000 rpm for 10 min at 4 °C, and collected the supernatants. We placed the supernatants were placed in a 96-well plate and mixed them with 10 µl of H_2_DCF-DC solutions, followed by incubation in the dark for 25 min. We measured the samples with a microplate reader (DTX800, Beckman Coulter, Austria) at the excitation wavelength 480 nm and the emission wavelength 530 nm.

### Investigation of lipid peroxidation (MDA assay)

Twenty-four h after reperfusion, we determined the MDA level using a calorimetric assay. MDA is the end-product of lipid hydroperoxide decomposition. In brief, we harvested the cerebral penumbra from each group and homogenized them in lysis buffer. The samples were mixed with 10 µl of butylated hydroxytoluene, 250 µl of 1 M phosphoric acid, and added 250 µl of 2-thiobarbituric acid. All reactions were mixed and incubated at 60 °C for 1 h. Then, the samples were centrifuged at 12,000 rpm for 5 min. The supernatants were transferred into a 96-well plate for measuring the absorbance by a microplate reader (BioTek Instruments Inc, Winooski, VT, USA) at 532 nm.

### MPO activity assay

MPO is a peroxidase enzyme that is most abundantly expressed in neutrophil granulocytes. Evaluating MPO activity is crucial to understanding its effects on inflammation. Briefly, the brain tissues of each group were collected from the ipsilateral hemisphere at 72 h after reperfusion. The MPO activity was measured following the myeloperoxidase (MPO activity assay kit) (Abcam, MA, USA).

### Caspase-3 activity assay

The assay is based on detection of cleavage of substrate DEVD-AFC, which is cleaved by caspase-3. Twenty-four h after reperfusion, we collected the cerebral penumbra from each group and homogenized the samples in lysis buffer. Then, we measured caspase-3 activity using an assay kit (Abcam, MA, USA).

### Western blot analysis

Twenty-four or seventy-two hours after reperfusion, cerebral penumbra of each group was harvested and stored them at − 80 °C until use. To extract total proteins, we homogenized the brains and determined the total protein concentrations using the Bradford protein assay (Bio-Rad Laboratories, Hercules, CA, USA) with bovine serum albumin (BSA) as the standard. We applied 25 µg of total protein in each sample were applied into the lanes of a 10–15% sodium dodecyl sulfate–polyacrylamide gel electrophoresis (SDS-PAGE) gels. Then, we transferred isolated proteins to polyvinylidene difluoride (PVDF) membrane (Immobilon-P, Millipore, Bedford, MA, USA) and blocked them in a fresh blocking buffer (containing 5% skim milk in 0.1% Tween-20 in Tris-buffered saline, pH 7.4) for 3 h at room temperature. Subsequently, the membranes were incubated with the primary antibodies (anti-TLR4, anti-NF-κB, anti-TNF-α, anti-IL-1β, anti-iNOS, anti-MMP-9, anti-caspase-3, anti-GFAP, anti-Iba1, anti-occludin, or anti-claudin) overnight at 4 °C. Next, the membranes were washed with Tris-buffer saline and Tween-20 (TBST) and incubated them with anti-rabbit IgG or anti-mouse peroxidase-conjugated secondary antibody. The membranes were incubated with Immobilon Western (Millipore, MA, USA) and exposed them to X-ray film. Densitometric analysis was performed by a scanning films with a densitometer the results were normalized using β actin by image J analysis.

### Statistical analysis

The data are presented as mean ± standard error of the mean (SEM). Statistical differences between the two groups were determined by using Student’s *t*-test. Other data were analyzed using a one-way analysis of variance (ANOVA), followed by Dunnett’s post hoc test. However, the data with non-normal distribution were analyzed using Kruskal–Wallis test. A *p* value < 0.05 was considered a statistically significant difference between experimental and control groups.

## Discussion

Cerebral I/R injury leads to the excessive production of ROS^27^, a phenomenon that can activate a cascade of pathophysiological processes, including oxidative stress, inflammation, and neuronal apoptosis^28–30^. A previous study demonstrated the anti-inflammatory and anti-oxidant activity of morin in a transient cerebral model. Morin acts as a powerful anti-inflammatory agent by suppressing activation of NF-ĸB and the expression levels of pro-inflammatory cytokines such as TNF-α, IL-1β, and iNOS^19^. However, its benefits toward preventing or ameliorating BBB disruption after cerebral I/R have not been reported. Hence, in the present study, we first investigated the ability of morin to attenuate BBB and cerebral damage following cerebral ischemia/reperfusion injury during the acute phase of rats subjected to MCAO and reperfusion injury. We focused on three different time points: pre-ischemia, during ischemia, and during reperfusion. Our results demonstrated that morin treatment had no effect on body temperature, oxygen saturation, body weight or heart rate in the rats. Further, morin decreased NF-ĸB activation, whereas TNF-α, IL-1β, and iNOS expression decreased. The major findings showed that morin inhibited NF-ĸB activation which, in turn, decreased the expression of proinflammatory cytokines. Moreover, morin suppressed MMP-9 expression, which improved BBB disruption by reducing TJ protein degradation and BBB permeability at pre-ischemia, during ischemia, and during reperfusion.

Cerebral ischemia leads to cerebral infarction via mechanisms that cause irreversible damages and neuronal cell death^31^. The mechanisms occur extremely rapidly in the ischemic core, and thus this area is difficult to protect. However, preservation of the penumbra by residual or collateral blood flow and salvaging the area can help to prevent the continued growth of the ischemic core or decrease the infarct size. Our results showed that at all time points morin treatment improved the neurological outcome and attenuated the cerebral infarct size. When cerebral perfusion is re-established (reoxygenation), a high oxygen surge occurs in the penumbra. This event leads to marked increase in ROS generation. Many studies have reported that a large quantity of ROS causes subsequent oxidative stress and neuronal cell injury^5–7^. Following the oxidative stress of cerebral ischemia, inflammation, BBB disruption, and apoptosis are induced. Therefore, suppression of oxidative stress might prevent neuronal injury in cerebral I/R. A decrease of ROS may reduce neuronal cells death by attenuating lipid peroxidation and oxidative stress. Many studies have reported that morin has strong antioxidant properties. Morin prevents lipid peroxidation, scavenges intracellular ROS, and protects the antioxidant system^32–34^. Moreover, a previous study reported that morin plays an antioxidant role in the MCAO model. Our results confirmed that morin-treated groups tends to decrease ROS production and reduce lipid peroxidation, as observed by the MDA level. These outcomes are related to the levels of Iba1 and GFAP expression, markers that represent microglia and astrocytes, respectively. This study showed that microglia and astrocyte were the source of ROS and oxidative stress in cerebral I/R injury.

ROS overexpression also triggers neuronal inflammation, which includes the contribution of TLR4. TLR4 is upregulated after cerebral I/R injury to recognize the intracellular components from neuronal cell death was known as DAMPs^35,36^, and activation of TLR4 leads to neuronal inflammation through an NF-ĸB signaling pathway^8,9^. NF-ĸB activation plays an important role in pro-inflammatory cytokine regulation, including TNF-α, IL-1β, and iNOS. Therefore, TLR4 inhibition downregulates activation of the NF-ĸB signaling pathway, in addition to reducing pro-inflammatory cytokines production. Our studies found that morin attenuated TLR4 expression and also decreased NF-ĸB activation, which is closely related to reducing TNF-α, IL-1β, and iNOS expression. Furthermore, MMP-9, a pro-inflammatory cytokine produced by NF-ĸB activation, is also released to induce BBB disruption^20^. MMP-9 directly degrades the extracellular matrix, basal membrane, and TJ proteins, such as occludin and claudin, all of which lead to BBB breakdown^37^. Subsequently, the BBB disruption allows neutrophil infiltration from the blood into the brain at 72 h after cerebral I/R^12^. However, previous research suggested that suppressing MMP-9 expression improves BBB disruption by reducing TJ protein degradation and BBB permeability in a cerebral I/R model^26,38^. Our results revealed that morin administration suppressed MMP-9 expression. In addition, morin decreased the degradation of TJ proteins, like occludin and claudin, and reduced the infiltration of neutrophils, correlated with decreased MPO activity at 72 h after reperfusion. These results, related to the Evans blue assay, showed that the leakage of Evans blue dye in morin-treated groups was significantly decreased compared with the vehicle group. These findings suggest that morin decreases BBB permeability and BBB leakage. Moreover, we further studied the ultrastructural changes of the BBB using TEM. We found that the cerebral microvascular was altered in morin-treated groups. Sham rats showed mostly vascular lumen alterations and the formation of microvilli. These results suggest that morin reduces inflammation and enhances BBB integrity after cerebral ischemia/reperfusion in the MCAO model.

Several death receptor ligands, such as TNF-α and IL-1β, can markedly induce neuronal apoptosis^31,39^. Previous studies demonstrated that apoptosis plays an important role in the pathogenesis of cerebral I/R and leads to cerebral infarction. Decreasing caspase-3 expression reduces apoptotic cells and cerebral infarction after MCAO. A previous study revealed that morin-treated rats subjected to MCAO showed caspase-3 downregulation^19^. In our study, morin treatment at each time point decreased caspase-3 expression and activity. In addition, we noted fewer TUNEL-positive cells in the ischemic brain of morin-treated groups, data that indicate a decrease in apoptotic cells. Our results also showed histological changes in the cerebral cortex and striatum. We found that the number of neuronal cells was decreased in the vehicle group, and neuronal cells exhibited pyknotic nuclei and overall shrinkage. Hence, our data indicated that morin can reduce apoptosis in the acute phase of cerebral ischemia/reperfusion.

In conclusion, our study provided evidence that morin has a neuroprotective effect on cerebral ischemia/reperfusion. It improved cerebral damage and neurological outcomes by attenuating oxidative stress, inflammation, and apoptosis. Here, we found that morin improved BBB disruption and enhanced BBB integrity.

## Supplementary information

Supplementary file 1.

Supplementary datasets.
