# Supplementary material for: Morin protects the blood–brain barrier integrity against cerebral ischemia reperfusion through anti-inflammatory actions in rats
Source: Sci Rep. 2020 Aug 7;10:13379. doi: 10.1038/s41598-020-70214-8 (PMC7414849; doi:10.1038/s41598-020-70214-8)
Supplement: Supplementary file 1 [file 41598_2020_70214_MOESM1_ESM.docx]

**Morin protects the blood-brain barrier integrity against cerebral ischemia reperfusion through anti-inflammatory actions in rats**

**Satchakorn Khamchai^1,2^, Wijittra Chumboatong^1^, Janejira Hata^3^, Chainarong Tocharus^4^, Apichart Suksamrarn^3^, Jiraporn Tocharus^1,*^,**

^1^Department of Physiology, Faculty of Medicine, Chiang Mai University, Chiang Mai, 50200, Thailand

^2^Graduate School, Chiang Mai University, Chiang Mai, 50200, Thailand

^3^Department of Chemistry and Center of Excellence for Innovation in Chemistry, Faculty of Science, Ramkhamhaeng University, Bangkok, 10240, Thailand

^4^Department of Anatomy, Faculty of Medicine, Chiang Mai University, Chiang Mai, 50200, Thailand

**^*^**Address for Correspondence:

Jiraporn Tocharus, PhD

Department of Physiology, Faculty of Medicine

Chiang Mai University

Chiang Mai 50200

Thailand

Telephone: 66 53945362

E-mail*:* [jtocharus@gmail.com](mailto:jtocharus@gmail.com)

**TLR4**

| **N1** | **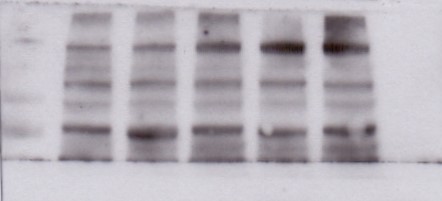**  89 kDa |
| --- | --- |
| **N2** | **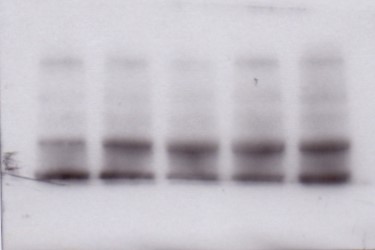**  89 kDa |
| **N3** | **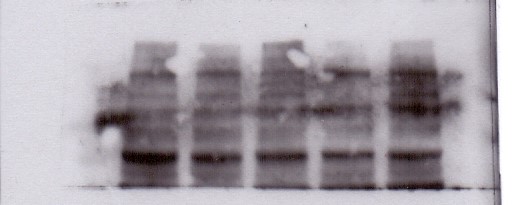**  89 kDa |

**NF-ĸB**

| **N1** | **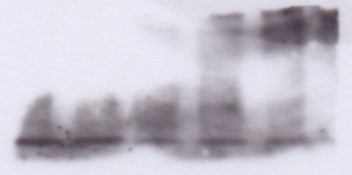**  65 kDa |
| --- | --- |
| **N2** | **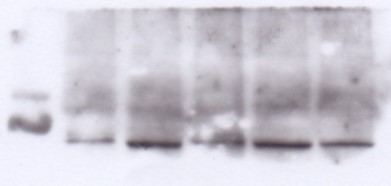**  65 kDa |
| **N3** | **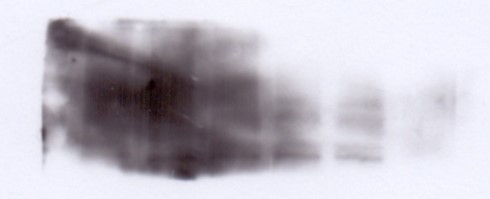**  65 kDa |

**TNF-α**

| **N1** | **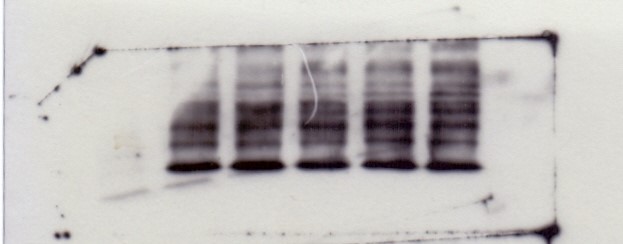**  17 kDa |
| --- | --- |
| **N2** | **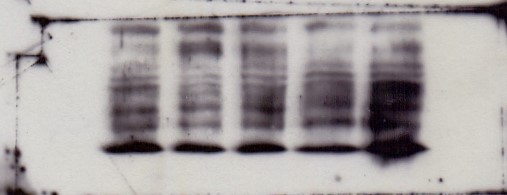**  17 kDa |
| **N3** | **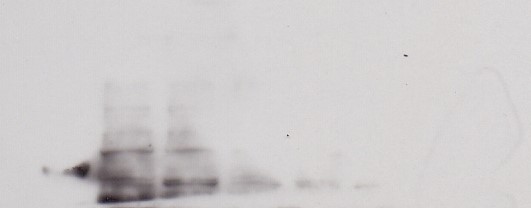**  17 kDa |

**IL-1β**

| **N1** | **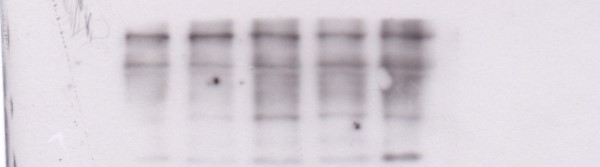**  17 kDa |
| --- | --- |
| **N2** | **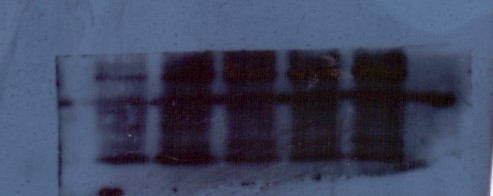**  17 kDa |
| **N3** | **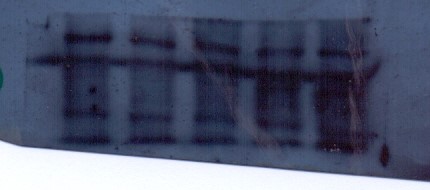**  17 kDa |

**iNOS**

| **N1** | **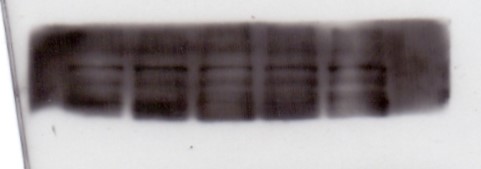**  130 kDa |
| --- | --- |
| **N2** | **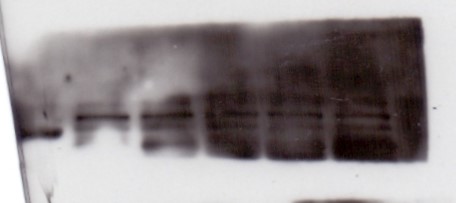**  130 kDa |
| **N3** | **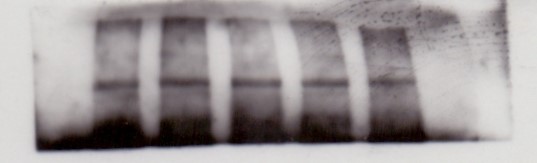**  130 kDa |

**MMP-9**

| **N1** | **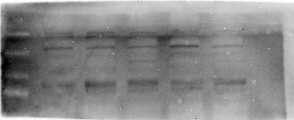**  84 kDa |
| --- | --- |
| **N2** | **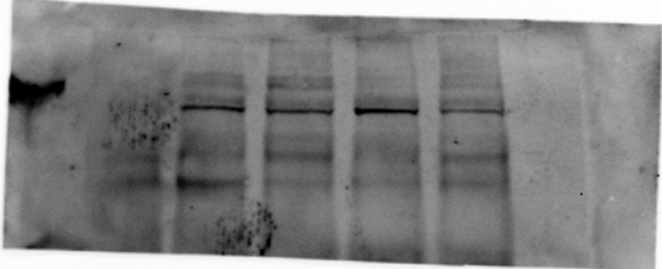**  84 kDa |
| **N3** | ****  84 kDa |

**Occludin**

| **N1** | **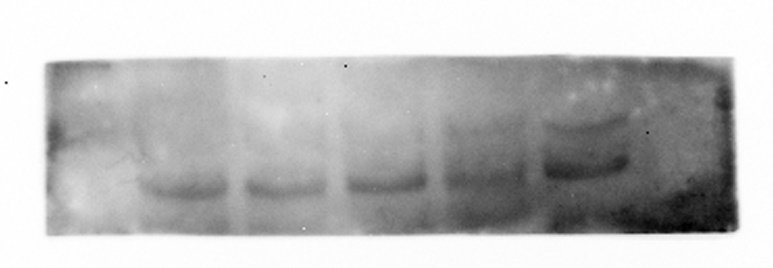**  55 kDa |
| --- | --- |
| **N2** | **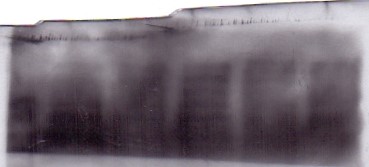**  55 kDa |
| **N3** | **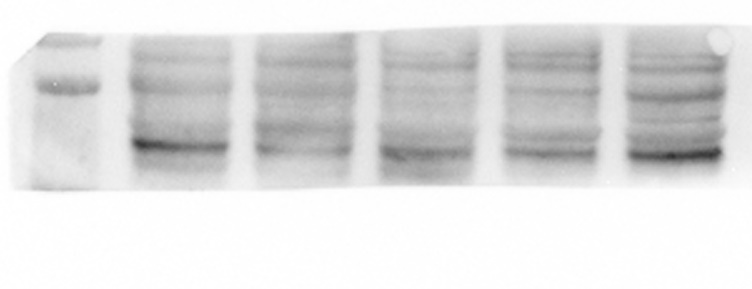**  55 kDa |

**Claudin**

| **N1** | **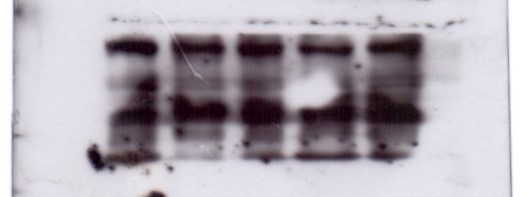**  17 kDa |
| --- | --- |
| **N2** | **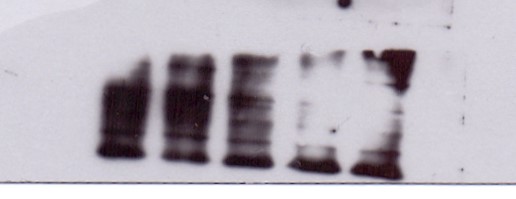**  17 kDa |
| **N3** | **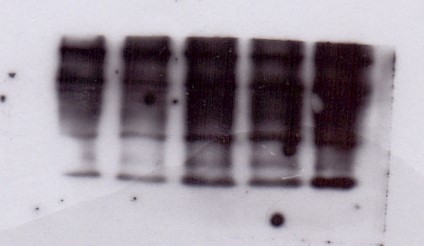**  17 kDa |

**Caspase-3**

| **N1** | **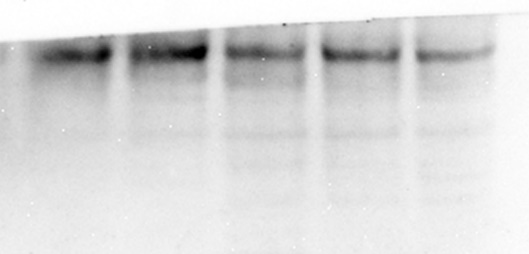**  17 kDa kDa |
| --- | --- |
| **N2** | **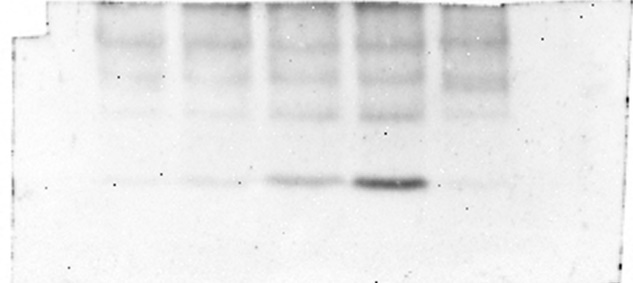**  17 kDa |
| **N3** | **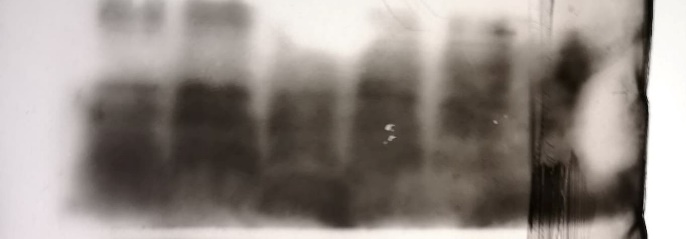**  17 kDa |

**GFAP**

| **N1** | **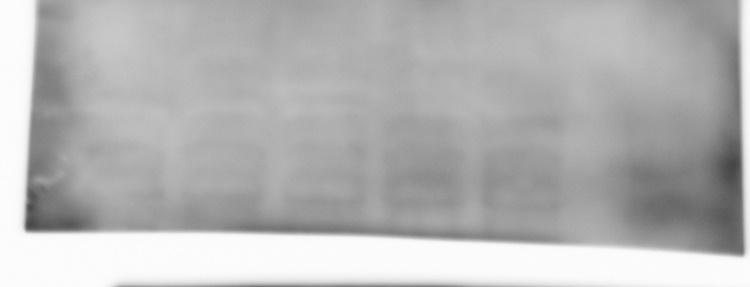**  50 kDa |
| --- | --- |
| **N2** | **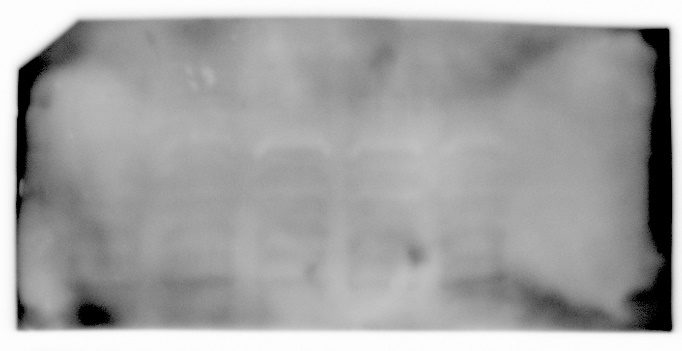**  50 kDa |
| **N3** | **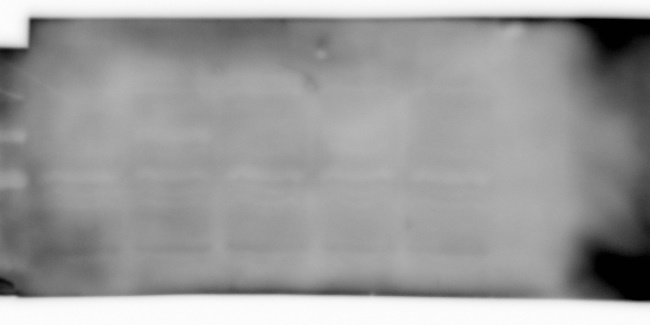**  50 kDa |

**Iba1**

| **N1** | **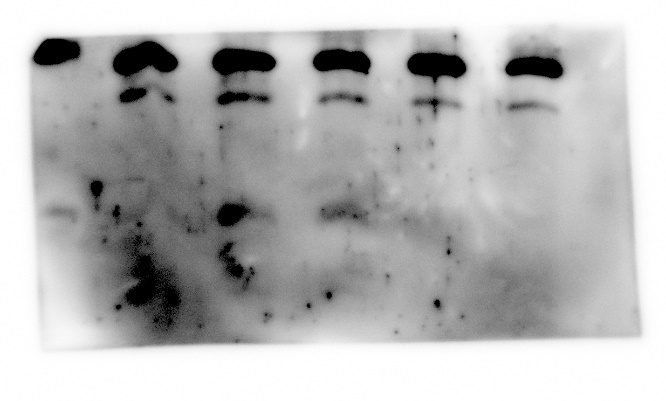**  17 kDa |
| --- | --- |
| **N2** | **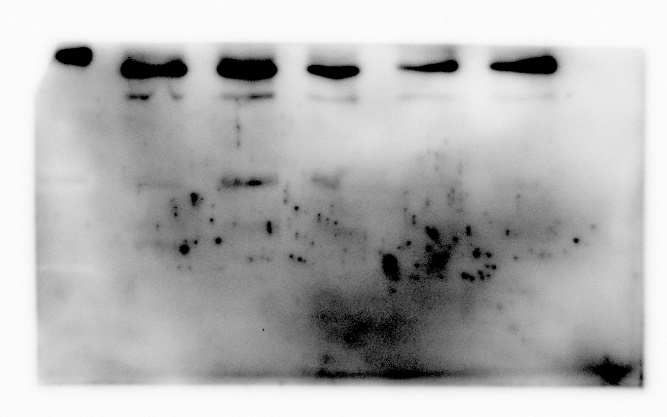**  17 kDa |
| **N3** | **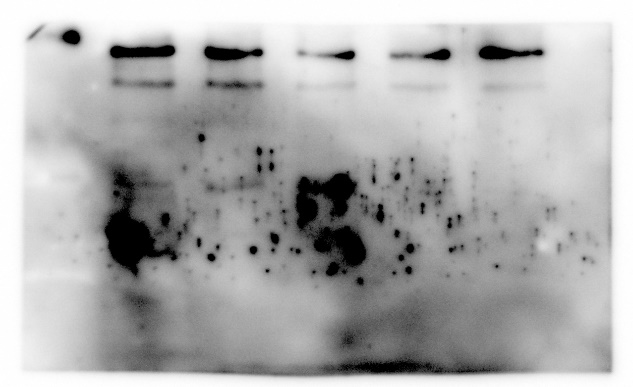**  17 kDa |

**Actin**

| **Figure 4** | **** |
| --- | --- |
| **Figure 5** | **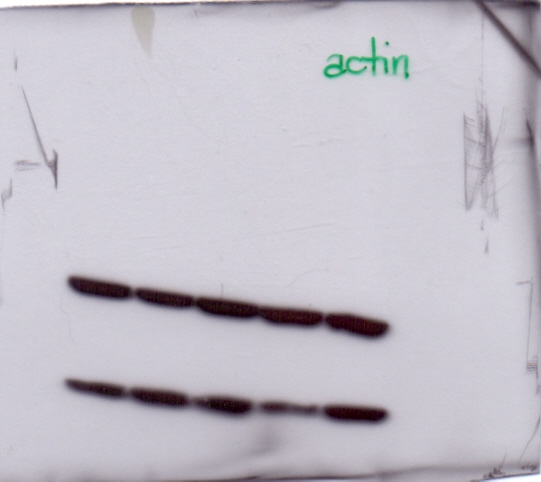** |
| **Figure 7** | **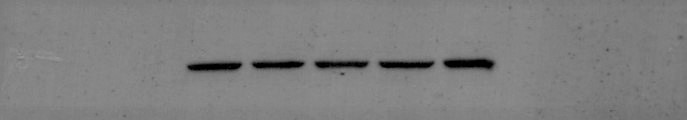** |
| **Figure 8** | **** |
